# Genome-wide association study and selective sweep analyses identify loci associated with plant height and spike number in a Chinese micro-core collection of common wheat (*Triticum aestivum* L.)

**DOI:** 10.3389/fpls.2026.2003113

**Published:** 2026-10-30

**Authors:** Xinqian Lyu, Dongli Lu, Zhenhao Zhu, Yiyi Tu, Fan Yang, Chengyun Li, Yangshan Hu

**Affiliations:** State Key Laboratory for Conservation and Utilization of Bio-Resources in Yunnan and College of Plant Protection, Yunnan Agricultural University, Kunming, Yunnan, China

**Keywords:** common wheat, landrace, plant height, spike number, genome-wide association study, quantitative trait locus, selective sweep analysis

## Abstract

Plant height (PH) and spike number per plant (SN) are important components of wheat architecture and adaptation. However, the extent to which loci underlying these traits overlap genomic regions targeted during modern breeding remains incompletely understood. We combined genome-wide association analysis with selective sweep scans to identify quantitative trait loci (QTLs) and positional candidate genes for these traits in common wheat. A panel of 280 accessions, comprising 133 landraces and 147 modern cultivars, was genotyped with a single-nucleotide polymorphism array, retaining 319,986 markers after quality control. Population analyses separated most landraces from modern cultivars and inferred migration edges from several landrace groups toward corresponding cultivar groups. Genome-wide association analyses of the two environments and best linear unbiased predictions identified 53 QTLs, comprising 18 for PH and 35 for SN. Of these, eight QTLs were detected in at least two datasets. Selective sweep scans based on nucleotide diversity, population differentiation, and the cross-population composite likelihood ratio identified genomic regions showing signatures of breeding selection. Three QTLs, *QPH.ynau-5B.1*, *QSN.ynau-1A.4*, and *QSN.ynau-6B.3*, overlapped these regions. Genes encoding a NAC domain-containing transcription factor, beta-glucanase, and diacylglycerol kinase were prioritized as positional candidates within the overlapping regions. These findings connect trait-associated loci with genomic signatures of breeding selection and provide candidate loci and genes for understanding and improving wheat architecture.

## 1 Introduction

Common wheat (*Triticum aestivum* L.) is a major staple crop worldwide, providing approximately 20% of the calories and protein consumed by the global population (Shiferaw et al., 2013). Given a growing population and climate-related constraints on wheat production, further elevating the yield potential of wheat has become a central objective to ensure global food security (Reynolds et al., 2009). Wheat grain yield per unit area is determined by spike density, grain number per spike, and grain weight. Spike number per plant (SN) reflects tillering and tiller fertility and contributes to spike density, although the relationship depends on planting density and crop management. Plant height (PH) is another important architecture-related trait associated with lodging susceptibility and biomass allocation (Peng et al., 1999; Slafer et al., 2014; Li et al., 2019). Deciphering the genetic basis of PH and SN is important for developing ideal-type cultivars with high yield and lodging resistance through molecular design (Wurschum et al., 2015).

During the “Green Revolution” in the mid-20th century, the semi-dwarfing alleles *Rht-B1b* and *Rht-D1b* were widely deployed in modern wheat breeding (Peng et al., 1999). These genes drastically reduced PH, significantly enhanced lodging resistance under high nitrogen conditions, and contributed substantially to increases in wheat yield (Hedden, 2003; Li et al., 2018). However, modern high-yield breeding relies not merely on the introgression of single dwarfing genes, but involves a simultaneous selection on canopy architecture, photosynthesis, and stem strength. Prolonged artificial directional selection not only alters the frequencies of target trait genes but also leaves selective sweep footprints on genomic regions that are physically linked or functionally coordinated (Walkowiak et al., 2020). Therefore, investigating the genome-wide selection experienced by modern improved cultivars relative to traditional landraces is essential for understanding the evolutionary trajectory of complex agronomic traits (Cavanagh et al., 2013).

Landraces are invaluable genetic resources preserved after long-term natural adaptation and early unconscious selection by farmers, harboring abundant variations for stress tolerance and broad adaptability (Lopes et al., 2015; Hu et al., 2023). In recent years, genome-wide association studies (GWAS) have emerged as the mainstream approach for discovering elite alleles for complex agronomic traits in wheat (Wu et al., 2025; Shang et al., 2026; Teku et al., 2026). Nevertheless, GWAS alone can only establish a static relationship between genotype and phenotype, falling short of revealing the dynamic selection history of alleles during population evolution. Integrating GWAS with population genomic selective sweep analysis can identify quantitative trait loci (QTLs) associated with target traits and prioritize positional candidate genes located in genomic regions showing signatures consistent with breeding selection. This combined approach may provide hypotheses regarding the genetic changes associated with the improvement of multiple traits (Hao et al., 2020; Sehgal et al., 2024).

In light of this, the present study assembled a natural association panel comprising 133 landraces and 147 modern cultivars and genotyped it using a 120K single nucleotide polymorphism (SNP) array. Using phenotypic data from two location-year environments, this study aims to: (1) characterize population genetic structure and putative historical gene flow; (2) identify reproducible QTLs associated with PH and SN through GWAS; (3) perform genome-wide selective sweep scans utilizing combined population genetic differentiation (*F*_ST_), nucleotide diversity ratio (π ratio), and cross-population composite likelihood ratio (XP-CLR) algorithms; and (4) identify positional candidate genes within regions where QTLs overlap selective sweep signals.

## 2 Materials and methods

### 2.1 Plant materials and phenotypic evaluation

The 280 wheat accessions, including landraces and modern cultivars, were provided by the Key Laboratory of Plant Genetics and Breeding of Sichuan Province, Sichuan Agricultural University, Chengdu, Sichuan, China. Field experiments were conducted during the 2024–2025 cropping season at Lancang (22.68°N, 99.79°E, altitude 1391.0 m) and the 2025–2026 cropping season at Yimen (24.54°N, 102.20°E, altitude 1389.4 m). The experimental design was a randomized complete block design with three replicates. Each plot was planted with a row length of 1.0 m, an inter-row spacing of 30 cm, and 20 plants per row. Field management followed local high-yield wheat cultivation practices. To ensure phenotypic accuracy and minimize edge effects, 10 representative plants from the center of each plot were randomly selected for trait evaluation. PH was measured as the distance from the soil surface to the tip of the spike (excluding the awns) at the maturity stage. SN was recorded by counting the number of spikes per plant. The mean values of the selected plants from each plot were used for subsequent genetic analyses.

### 2.2 Phenotypic data analysis and heritability estimation

Phenotypic data across the 2025 and 2026 environments were analyzed using R software. Best linear unbiased prediction (BLUP) values for PH and SN across environments were estimated using the lme4 package in R to account for environmental and design effects (Bates et al., 2015). Broad-sense heritability (*H^2^*) was calculated based on the variance components using the formula (Shahinnia et al., 2022):


$$H^{2}=\frac{\sigma_{G}^{2}}{\sigma_{G}^{2}+\frac{\sigma_{e}^{2}}{T}}$$

where $\sigma_{G}^{2}$ and $\sigma_{e}^{2}$ represent genetic and residual variances, respectively. T represents the number of trials.

### 2.3 Genotyping and quality control

Genomic DNA was extracted from young leaves using the cetyltrimethylammonium bromide method. Genotyping was performed using the Wheat 120K SNP array (Tcuni Inc., Chengdu, China). Raw SNP data were subjected to quality control using PLINK v1.9 (Purcell et al., 2007). SNPs with minor allele frequency (MAF) < 0.05, missing rate > 10%, and heterozygosity > 10% were filtered out, resulting in a final set of 319,986 high-quality SNPs for downstream analyses. The polymorphism information content (PIC) was calculated according to the method as follows (Serrote et al., 2020):


$$PIC=1-(\sum_{i=1}^{n}p_{i}^{2})-\sum_{i=1}^{n-1}\sum_{j=i+1}^{n}2p_{i}^{2}p_{j}^{2}$$

where $p_{i}$ and $p_{j}$ represent the frequencies of the *i*-th and *j*-th alleles in the population, respectively, and *n* is the number of alleles at a given locus.

The Shannon-Wiener index (SWI) was calculated using the following formula (Hill, 1973):


$$SWI=-\sum_{i=1}^{n}p_{i}\text{ln}(p_{i})$$

where $p_{i}$ is the frequency of the *i*-th allele and *n* denotes the total number of alleles at that locus.

### 2.4 Population structure, linkage disequilibrium, and gene introgression

To decipher the genetic relationships, a phylogenetic tree was constructed using IQ-TREE software based on the maximum likelihood (ML) method (Nguyen et al., 2015), and visualized and annotated using the Interactive Tree Of Life (iTOL) (Letunic and Bork, 2019). Population structure was inferred using the ADMIXTURE v1.3.0 software, testing hypothetical subpopulation numbers (K) ranging from 2 to 9 (Alexander et al., 2009). The optimal K value was strictly determined by identifying the minimum cross-validation (CV) error. Additionally, principal component analysis (PCA) was performed using PLINK v1.9 based on genome-wide SNPs to assess population structure (Purcell et al., 2007). The identity by state (IBS) genetic similarity heatmap was visualized using the R package pheatmap, and PCA scatter plots were generated using the R package ggplot2. The linkage disequilibrium (LD) decay was evaluated using PopLDdecay software, and the LD decay distance was estimated when the r^2^ value dropped to 0.2 (Zhang et al., 2019). To investigate historical gene flow and introgression among landraces and modern cultivars, we utilized TreeMix v1.13 (Pickrell and Pritchard, 2012). Migration events were incrementally added, and the optimal number of migration edges was determined based on the variance explained by the model.

### 2.5 GWAS

GWAS was conducted for PH and SN across individual environments and BLUP values using GEMMA v0.98.6 (Zhou and Stephens, 2012). To account for population structure and minimize false positives, a mixed linear model was employed incorporating the first three principal components as fixed covariates to account for population structure. A genomic relatedness matrix was included to model the random polygenic effect and account for relatedness among accessions. Marker associations were evaluated using the Wald test, with a nominal threshold of *P* ≤ 1 × 10^-4^. To reduce redundant association signals, SNPs meeting this threshold in at least one annual or BLUP dataset were subjected to forward conditional analysis. Analyses were conducted separately for each trait and chromosome, with selected SNP dosages successively included as additional fixed covariates alongside the principal components. Each selected SNP was subsequently reassessed while conditioning on the other selected SNPs on the same chromosome. Signals were retained if their conditional *P* value remained ≤ 1 × 10^-4^ in at least one dataset. The retained signals were designated as QTLs, and their selected SNPs were used as representative markers. Manhattan and quantile–quantile plots were generated in R.

### 2.6 Genomic diversity and selective sweep analysis

To identify genomic regions subjected to human selection during modern wheat breeding, we performed a genome-wide selective sweep analysis by comparing the modern cultivar population against the landrace population. π ratio (π_landrace/π_cultivar) and *F*_ST_ were calculated using VCFtools with a sliding window approach (window size = 1000 kb, step size = 500 kb) (Danecek et al., 2011). The XP-CLR statistic was computed to detect allele frequency differentiation over linked loci (window size = 1000 kb, step size = 500 kb, minimum SNPs per window = 5, correlation level = 0.95) (Chen et al., 2010). Selective sweep intervals were defined by identifying regions located in the top 5% of the empirical distributions.

### 2.7 Candidate gene prediction and colocalization analysis

Candidate genes were predicted by intersecting the QTLs with the identified selective sweep intervals. Gene annotations within these co-localized regions were extracted based on the reference genome International Wheat Genome Sequencing Consortium (IWGSC) RefSeq v2.1 annotation database (https://www.wheatgenome.org/projects/reference-genome-project/refseq-v2.1).

## 3 Results

### 3.1 Geographical distribution, genomic variation, and population structure of wheat panel

To explore the genetic diversity of the wheat population, an association panel comprising 280 wheat accessions was assembled in this study. Based on their breeding history and origins, these accessions were primarily categorized into 147 modern cultivars and 133 landraces, with geographic origins covering a broad range of ecological regions, including Huang-Huai-Hai winter wheat region (HHHW), Middle and Lower Yangtze River Valley winter wheat region (MLYW), North-Eastern spring wheat region (NES), Northern spring wheat region (NS), Northern winter wheat region (NW), North-Western spring wheat region (NWS), Qinghai-Xizang spring and winter wheat region (QXSW), South China winter wheat region (SCW), South-Western winter wheat region (SWW), Xinjiang spring and winter wheat region (XSW) and 16 wheat accessions introduced from Chile, Japan, Italy, Mexico, Romania, Ukraine, and the United States (**Figure 1A**). All accessions were genotyped using the 120K SNP array. Owing to the characteristic cross-capture of probe sequences across homoeologous groups in allohexaploid wheat, individual probes can interrogate multiple homoeologous loci simultaneously, yielding more detectable markers than the nominal probe count. Following quality filtering, a total of 319,986 high-quality SNP markers were retained for downstream genetic analyses. The distribution of these SNPs across the three wheat sub-genomes was uneven (**Figure 1B**). The B sub-genome harbored the highest number of SNPs, followed by the A sub-genome, whereas the D sub-genome contained the fewest SNPs. In addition to marker abundance, we evaluated the genetic diversity indices for each sub-genome. At the whole-genome level, the average MAF, observed heterozygosity (Ho), expected heterozygosity (He), and PIC were 0.2360, 0.0463, 0.3251, and 0.2636, respectively. Among the three sub-genomes, the A and B sub-genomes displayed higher genetic diversity, while the D sub-genome showed a significantly restricted diversity level (**Table 1**). The lower diversity of the D sub-genome is consistent with its known demographic bottleneck.

**Figure 1:**
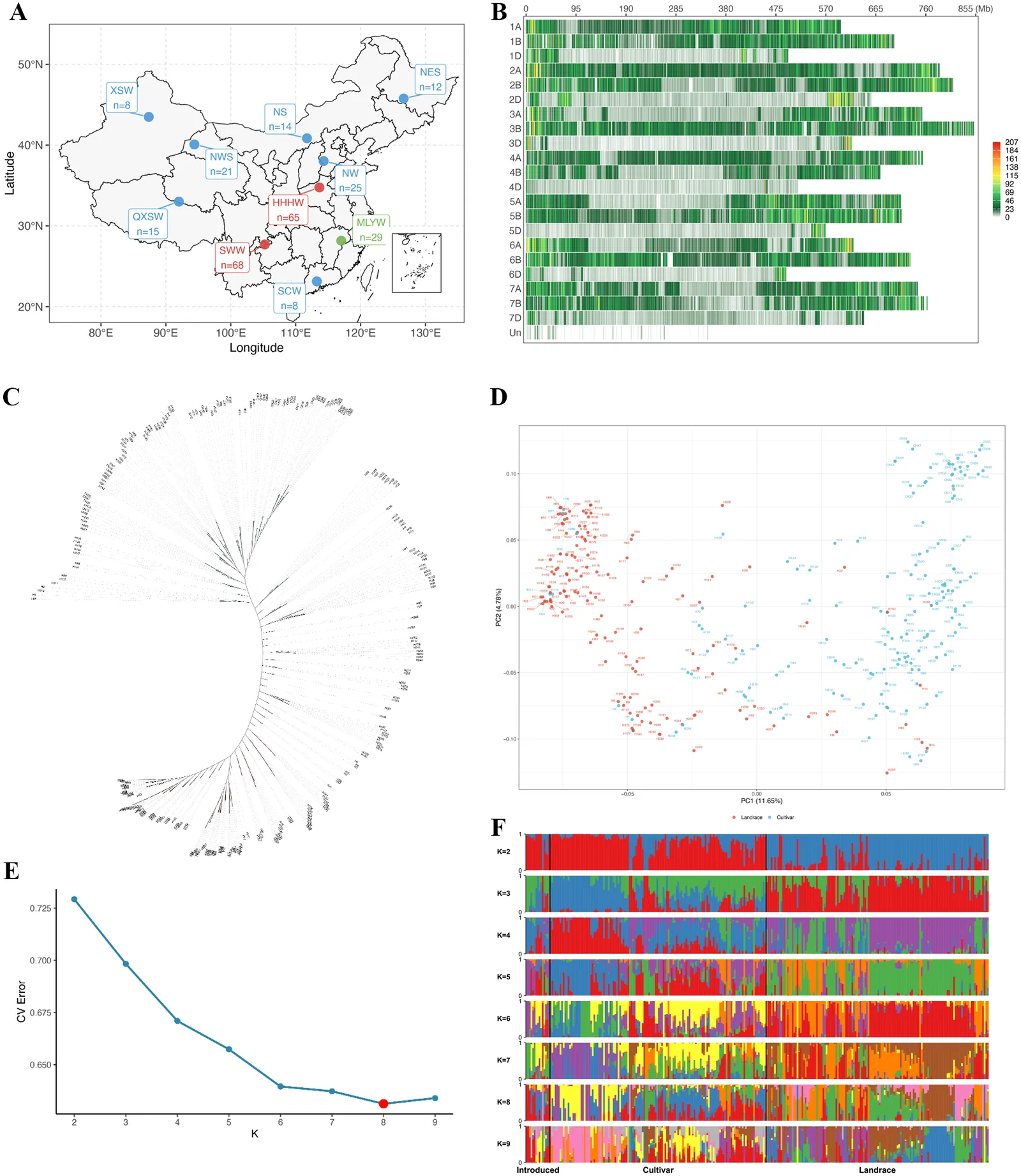
Geographic distribution, genome-wide variation, and population structure of the wheat panel. **(A)** Geographic origins of the common wheat accessions. **(B)** Density of 319,986 high-quality single-nucleotide polymorphism (SNP) markers along the 21 chromosomes; the color scale represents the number of SNPs in each genomic interval. **(C)** Maximum-likelihood phylogenetic tree based on genome-wide SNPs. **(D)** Principal component analysis (PCA), with colors distinguishing landraces and modern cultivars; PC1 and PC2 explain 11.65% and 4.78% of the genetic variation, respectively. **(E)** ADMIXTURE cross-validation (CV) error for K = 2–9; the red point marks the minimum CV error at K = 8. **(F)** ADMIXTURE ancestry proportions for K = 2–9. Each vertical bar represents one accession, colors indicate inferred ancestry components, and accessions are ordered as introduced accessions, modern cultivars, and landraces. HHHW, Huang-Huai-Hai winter wheat region; MLYW, Middle and Lower Yangtze River Valley winter wheat region; NES, North-Eastern spring wheat region; NS, Northern spring wheat region; NW, Northern winter wheat region; NWS, North-Western spring wheat region; QXSW, Qinghai-Xizang spring and winter wheat region; SCW, South China winter wheat region; SWW, South-Western winter wheat region; XSW, Xinjiang spring and winter wheat region.

**Table 1:** Statistics of genetic diversity of SNP markers across the whole genome and the sub-genomes.

Sub-genome	SNPs	Proportion (%)	MAF	Ho	He	PIC	SWI
A	126,891	39.66	0.2451	0.0473	0.3330	0.2688	0.5048
B	151,712	47.41	0.2420	0.0467	0.3315	0.2680	0.5033
D	41,022	12.82	0.1860	0.0414	0.2770	0.2311	0.4393
Un	361	0.11	0.2175	0.0474	0.3079	0.2516	0.4749
Whole genome	319,986	100.00	0.2360	0.0463	0.3251	0.2636	0.4957

MAF, minor allele frequency; Ho, observed heterozygosity; He, expected heterozygosity; PIC, polymorphism information content; SWI, Shannon-Wiener index.

Based on the high-quality SNP markers, an ML phylogenetic tree of the population was constructed. The result demonstrated that all accessions were primarily clustered into two distinct major clades, corresponding to the landraces and the modern cultivars (**Figure 1C**). PCA was consistent with the phylogenetic tree, where the first two principal components could separate the landraces from the modern cultivars (**Figure 1D**). This indicates that clear genetic differentiation has occurred at the genomic level between landraces and modern cultivars under long-term natural evolution and artificial selection. CV error analysis using ADMIXTURE indicated that the CV error reached its absolute minimum at K = 8, implying the existence of finer genetic sub-structures within the population (**Figure 1E**). However, at K = 3, the population structure plot reflected the major genetic background divergence among introduced accessions, modern cultivars, and landraces (**Figure 1F**). The structure analysis showed some genomic introgression and gene flow between modern cultivars and introduced accessions, while landraces maintained relatively ancient and independent genetic components. Furthermore, the IBS kinship matrix (**Supplementary Figure S1**) also showed similarity among accessions within the same main group.

### 3.2 Principal component clustering, linkage disequilibrium, and TreeMix analysis

To classify the population structure more objectively and quantitatively, a K-means clustering algorithm was applied to the PCA results. Compared to traditional prior classifications based on pedigree or geographical origins, K-means clustering independently identifies optimal grouping boundaries in multi-dimensional space through mathematical modeling. The clustering results further confirmed the significant genetic separation between the landraces and the modern cultivars (**Figure 2A**). The decay rate of LD is a metric that determines the required marker density and mapping resolution in genome-wide association studies (GWAS), while also reflecting the historical recombination and selection pressure of the population. We evaluated the genome-wide LD decay trend of the panel. The results demonstrated that as the physical distance along the chromosomes increased, the linkage disequilibrium coefficient (r^2^) between alleles decayed rapidly. The distance at which r^2^ declined to 0.2 was approximately 3164.7 kb (**Figure 2B**). This relatively long LD extent may reflect population structure, demographic history, and breeding selection.

**Figure 2:**
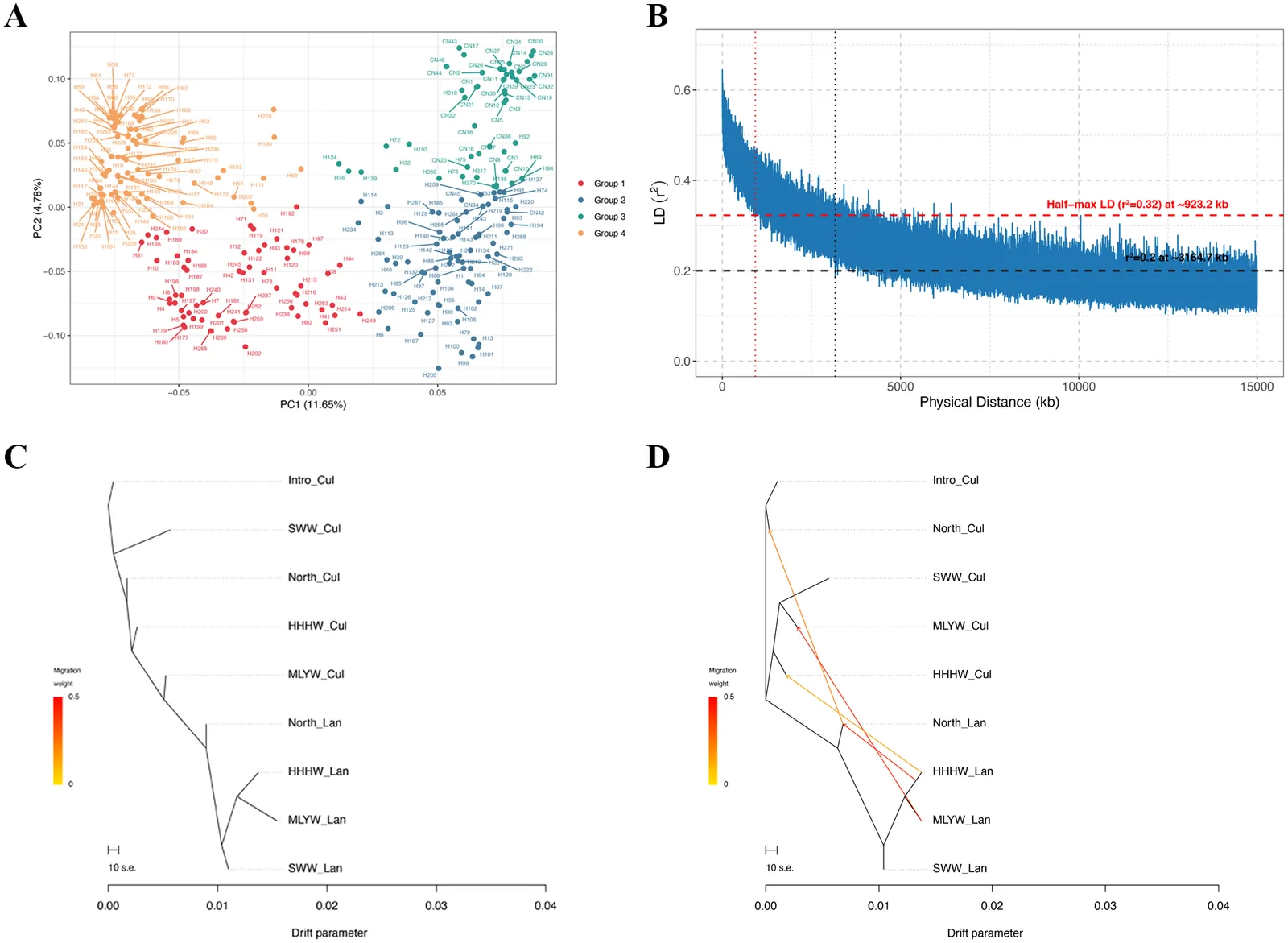
Population clustering and genome-wide linkage disequilibrium decay. **(A)** K-means clustering based on genome-wide principal component analysis. Each point represents one wheat accession, and colors indicate the four clusters; PC1 and PC2 explain 11.65% and 4.78% of the genetic variation, respectively. **(B)** Genome-wide linkage disequilibrium (LD) decay. The blue curve shows the change in the LD coefficient r^2^ with physical distance. The red dashed lines indicate half-maximal LD (r^2^ = 0.32), corresponding to approximately 923.2 kb, and the black dashed lines indicate r^2^ = 0.20, corresponding to an LD decay distance of approximately 3,164.7 kb. **(C)** TreeMix population tree under a model with no migration events (m = 0). The horizontal scale denotes the genetic drift parameter. **(D)** TreeMix population tree with four migration events (m = 4). Colored arrows indicate inferred migration directions, with color representing migration weight. The major migration edges indicate gene flow from the MLYW, North, and HHHW landrace groups to the corresponding modern cultivar groups. HHHW, Huang-Huai-Hai winter wheat region; MLYW, Middle and Lower Yangtze River Valley winter wheat region; Lan, landrace; Cul, modern cultivar.

To examine the historical relationships among the subpopulations, we constructed population graphs using TreeMix. Under the model without migration edges (*m* = 0), the landrace and modern cultivar subpopulations were broadly separated into two major clades (**Figure 2C**), consistent with the differentiation observed in the PCA. However, the residual covariance pattern indicated that a strictly bifurcating tree did not fully explain the relationships among the sampled subpopulations. After allowing four migration edges (*m* = 4), TreeMix inferred migration edges connecting branches associated with the MLYW, North, and HHHW landrace groups associated with the corresponding modern cultivar groups (**Figure 2D**).

### 3.3 Phenotypic variation and GWAS of PH and SN

To investigate the genetic basis of PH and SN, we evaluated the phenotypic variation of the association panel across two years and calculated their BLUP values. The results revealed significant phenotypic divergence between landraces and modern cultivars for both agronomic traits (**Figure 3A**). Landraces exhibited significantly higher average PH and SN compared to modern cultivars. Phenotypic correlation analysis demonstrated significant positive correlations for PH and SN across different environments (**Figure 3B**). Further calculation of broad-sense heritability (H^2^) revealed that the heritability for PH and SN reached 90.69% and 79.98%, respectively.

**Figure 3:**
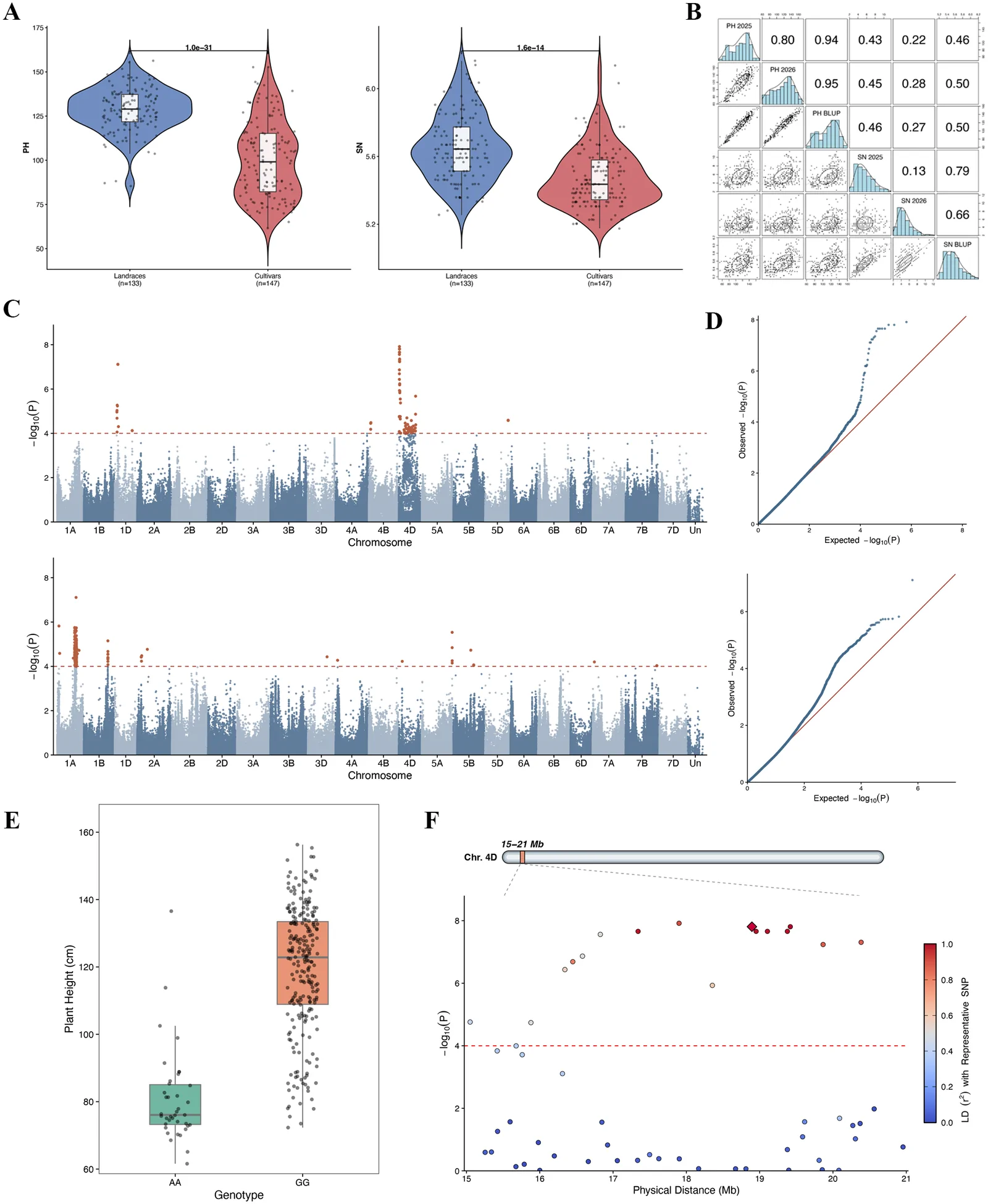
Phenotypic variation and genome-wide associations for plant height and spike number. **(A)** Distributions of best linear unbiased prediction (BLUP) values for plant height (PH) and spike number per plant (SN) in landraces (n = 133) and modern cultivars (n = 147). Points represent individual accessions, boxes show medians and interquartile ranges, and P values are shown above the comparisons. **(B)** Pairwise correlation matrix for PH and SN measured in 2025 and 2026 and their BLUP values. Diagonal panels show distributions, lower-triangle panels show pairwise scatterplots, and upper-triangle panels show correlation coefficients. **(C)** Manhattan plots for PH (upper) and SN (lower) based on BLUP values. Chromosomes are shown in alternating colors; red dashed lines indicate *P* = 10^-4^. **(D)** Quantile–quantile (QQ) plots for the GWAS of PH (upper) and SN (lower) based on BLUP values. Blue points represent the observed versus expected −log_10_(*P*) values, and the red diagonal indicates the expected distribution. **(E)** Allelic effect of the chromosome 4D peak SNP *4D_18894112*. Points represent individual accessions. **(F)** Regional association plot for the 15–21 Mb interval on chromosome 4D. The diamond denotes the peak SNP, and the remaining SNPs are colored according to their linkage disequilibrium (r^2^) with the peak SNP.

Based on the 319,986 SNP markers obtained previously, we performed GWAS for PH and SN across the three datasets. The Manhattan and quantile-quantile (QQ) plots for the BLUP data are highlighted in the main text as representatives (**Figures 3C, D**). All QQ plots indicated good model fitness, effectively controlling for false-positive inflation arising from population structure (**Supplementary Figure S2**). Following conditional association analysis, 53 QTLs were retained, comprising 18 for PH and 35 for SN (**Supplementary Table S1**). We further filtered for stable QTLs consistently detected in at least two datasets. As a result, 8 stable QTLs were reproducibly detected (**Table 2**), representing association signals for PH and SN in this population.

**Table 2:** Stable genome-wide QTLs for PH and SN.

Trait	QTL	Datasets	Peak SNP position	*P*_wald	MAF	BETA	PVE (%)
PH	*QPH.ynau-1D.1*	2025, 2026, BLUP	1D_67562322	2.51 × 10^-8^	0.073	-12.92	6.35
PH	*QPH.ynau-4D.1*	2025, 2026, BLUP	4D_18894112	3.87 × 10^-9^	0.143	-10.986	8.21
PH	QPH.ynau-4D.2	2025, 2026, BLUP	4D_389939803	2.10 × 10^-6^	0.280	6.294	5.84
PH	*QPH.ynau-5D.1*	2026, BLUP	5D_532684498	2.55 × 10^-5^	0.119	-7.725	4.64
SN	*QSN.ynau-1A.2*	2026, BLUP	1A_39056365	1.50 × 10^-6^	0.080	0.107	7.68
SN	*QSN.ynau-1A.4*	2026, BLUP	1A_433811958	7.75 × 10^-8^	0.330	-0.096	18.07
SN	*QSN.ynau-2A.2*	2026, BLUP	2A_234772036	1.06 × 10^-7^	0.052	1.295	11.30
SN	*QSN.ynau-7B.2*	2026, BLUP	7B_722662852	3.17 × 10^-7^	0.085	0.990	9.65

PH, plant height; SN, spike number per plant; MAF, minor allele frequency; BETA, estimated additive effect per copy of the non-reference allele; PVE, descriptive estimate of the percentage of phenotypic variance explained by each peak SNP. Peak SNP identifiers follow the chromosome_position format, with physical positions expressed in base pairs according to the IWGSC RefSeq v2.1 assembly.

Among all detected loci, the most prominent signal for PH was located on chromosome 4D and designated *QPH.ynau-4D.1*. The peak marker, *4D_18894112*, was located at 18,894,112 bp on chromosome 4D and showed a significant association with PH. Genotype effect analysis based on the peak SNP revealed that accessions carrying the *AA* genotype had a significantly reduced PH compared to those carrying the *GG* genotype (*P* = 3.40 × 10^-23^, **Figure 3E**). To dissect this major locus, we generated a regional zoom plot for the 15–21 Mb interval, which illustrated the local linkage disequilibrium decay pattern surrounding the peak SNP (**Figure 3F**). The candidate interval of 15–21 Mb encompasses *Rht-D1* (*TraesCS4D03G0067100*; 19,189,660–19,191,531 bp), which is located approximately 295.5 kb from the peak marker. Two nearby markers, *4D_17900162* and *4D_18951612*, exhibited strong LD with the peak marker (r^2^ = 0.905 and 0.993, respectively). These local association and LD patterns support *Rht-D1* as a positional candidate underlying *QPH.ynau-4D.1*.

### 3.4 Genome-wide selective sweep analysis and integration with GWAS

Genome-wide selective sweep analysis comparing landraces with modern cultivars identified candidate regions showing signatures of selection using π ratio, *F*_ST_, and XP-CLR (**Figure 4A**). Most genes within these regions were located in the A and B sub-genomes, whereas the D sub-genome contained the fewest, which is consistent with its overall lower baseline of genetic diversity. Furthermore, intersection analysis based on homoeologous gene groups revealed that 60 homoeologous genes were located within candidate selected regions in all three sub-genomes (**Figure 4B**). Functional annotation included predicted roles in transcriptional regulation, carbohydrate metabolism, and cell wall-related processes (**Supplementary Table S2**).

**Figure 4:**
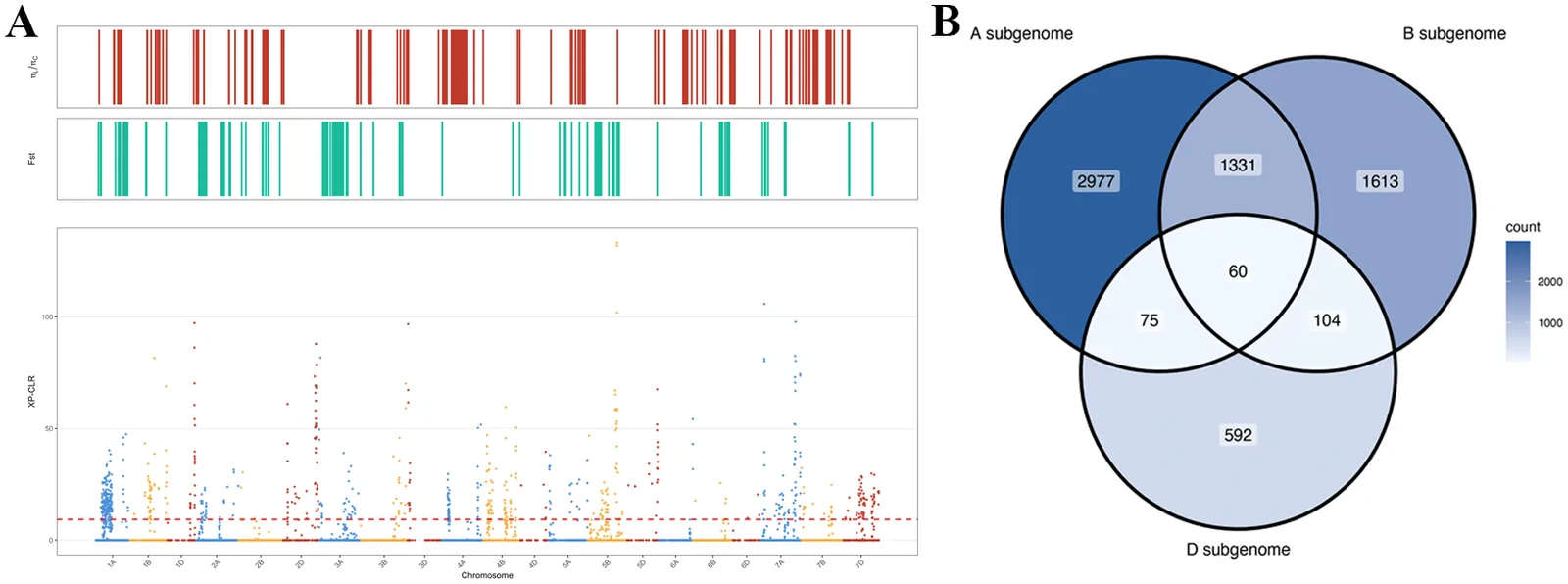
Genome-wide selective sweeps and selected genes across wheat sub-genomes. **(A)** Genome-wide selective sweep analysis comparing modern cultivars with landraces. From top to bottom, the tracks show signals identified using the nucleotide diversity ratio (π-landrace/π-cultivar), the population differentiation index (*F_ST_*), and the cross-population composite likelihood ratio (XP-CLR). Analyses used 1000-kb windows with 500-kb steps, and candidate sweep intervals were defined from the top 5% of the empirical distributions for all three statistics. **(B)** Venn diagram of selected genes in the A, B, and D sub-genomes. Numbers indicate genes unique to or shared among the sub-genomes.

To prioritize positional candidate genes potentially associated with PH and SN and located within genomic regions showing signatures of breeding selection, we strictly cross-referenced the 53 QTL intervals identified by GWAS with the candidate selected genes identified by the selective sweep analysis. The results revealed that three QTLs overlapped with the artificial selection signal regions. Among the loci controlling PH, one QTL exhibited strong selection signatures. Specifically, the locus *QPH.ynau-5B.1* interval on chromosome 5B contained an NAC domain-containing transcription factor (*TraesCS5B03G0893200*) and a Histone H4 variant (*TraesCS5B03G0893500*). Among the loci associated with SN, a total of two QTLs fell within the selective sweep intervals. Notably, the *QSN.ynau-1A.4* interval on chromosome 1A encompassed genes located within candidate sweep regions encoding beta-glucanase (*TraesCS1A03G0608300*) and diacylglycerol kinase (*TraesCS1A03G0610900*). Additionally, the locus *QSN.ynau-6B.3* also physically overlapped with a candidate selected region.

## 4 Discussion

### 4.1 Population structure and inferred historical gene flow

Deciphering the population genetic structure and historical gene exchange of crops is fundamental to understanding their domestication and breeding trajectories. The phylogenetic tree and PCA based on genome-wide SNPs revealed a significant genetic divergence between wheat landraces and modern cultivars. This pronounced genetic differentiation reflects the reshaping of the population genome by intense artificial directional selection aimed at high yield and broad adaptability during modern breeding (Hao et al., 2020; Liu et al., 2023). Introgression analysis based on TreeMix identified four inferred migration edges. We observed migration edges consistent with gene flow from landraces to modern cultivars. In modern wheat breeding practices, breeders have used landraces as parental material to optimize the local adaptation of introduced varieties (Wang et al., 2021; Zhou et al., 2021; Sadaat et al., 2026). Furthermore, we observed gene exchange networks among certain landrace subpopulations. This internal gene flow among landraces likely reflects historical seed exchange or natural hybridization facilitated by geographical proximity, promoting the sharing of elite genetic variations (Lopes et al., 2015). Regarding genome-wide diversity, the D sub-genome exhibited substantially lower variation density and fewer selected genes compared to the A and B sub-genomes. This is consistent with the genetic bottleneck experienced by the D genome donor (*Aegilops tauschii*) during the polyploidization of common wheat (Jia et al., 2013; Cavalet-Giorsa et al., 2024; Wright et al., 2024).

### 4.2 Genetic variation associated with PH and SN

PH and SN are core agronomic traits determining wheat canopy structure, lodging resistance, and ultimate yield (Hu et al., 2017; Ren et al., 2018; Li et al., 2022; Wang et al., 2025). In this study, the broad-sense heritabilities for these traits reached 90.69% and 79.98%, respectively, suggesting a substantial genetic contribution to variation within the tested population and environments (Juliana et al., 2019). Interestingly, our phenotypic data revealed that landraces had significantly higher SN than modern cultivars. This phenotypic difference may reflect a shift in yield-related plant architecture and tillering strategy over the course of wheat breeding. Landraces were shaped by natural and farmer-mediated selection under low-input, extensive, and variable agricultural conditions with relatively low planting densities. Under these traditional farming systems, strong tillering ability and high spike production represented a key adaptive strategy: this trait helped wheat compete with weeds, cope with unfavorable environmental conditions, and guarantee reproductive success for individual plants (Lai et al., 2025). Even so, landraces with abundant tillers generally produce smaller spikes, with fewer spikelets and grains per ear (Shan and Osborne, 2024). By comparison, modern high‑yield breeding for high‑density and high‑fertilizer environments favors the large‑spike ideotype, with selection focused on larger spikes, more fertile florets, and higher grain number per spike (Donald, 1968; Slafer et al., 2014; Li et al., 2022). Within dense stands, abundant or late‑emerging tillers behave as non‑productive sinks. They consume photosynthates during stem elongation yet frequently fail to produce heads, or generate poorly filled, sterile spikes (Duggan et al., 2005). For this reason, artificial selection in modern breeding has acted to reduce redundant tillers and improve tiller efficiency. Assimilates are then allocated preferentially to the main stem and early primary tillers, generating more uniform, vigorous spikes with better grain filling (Shan and Osborne, 2024; Lai et al., 2025). This trade‑off between per‑plant spike number and individual spike size illustrates how breeding has transformed wheat: selection moved away from the vegetative compensation strategy optimized for individual plants, toward a population‑level sink‑efficiency design suited for high‑density cultivation (Slafer et al., 2014; Li et al., 2022). We identified a total of 53 QTLs genome-wide, further refining 8 stable QTLs. Among all identified loci, the most prominent was the QTL controlling PH on chromosome 4D (*P* = 3.87 × 10^-9^). Its physical location and negative estimated allelic effect align with the cloned “Green Revolution” dwarfing gene, *Rht-D1* (Peng et al., 1999). The introduction of such dwarfing mutant alleles in the mid-20th century substantially enhanced stem lodging resistance under high nitrogen fertilizer applications and significantly increased the harvest index (Hedden, 2003).

### 4.3 Comparison of identified QTLs with previous studies

We compared the 53 QTLs with published loci, focusing on the eight detected in at least two datasets. For PH, *QPH.ynau-4D.1* lies within the PH QTL-rich cluster *QRC-4D-I*, which contains *Rht-D1* (Xu et al., 2023). *QPH.ynau-5D.1*, with its peak at 532.684 Mb on RefSeq v2.1, lies approximately 62.7 kb from *Xwmc640a*. This marker, located at 532.622 Mb, is the closest marker reported for *QHt.crc-5D*, suggesting that *QPH.ynau-5D.1* may correspond to this previously identified PH QTL (Huang et al., 2006; Xu et al., 2023). After conversion to RefSeq v1.0, the peak of *QPH.ynau-1D.1* mapped to 64.345 Mb. It lies 7.59 Mb from *BS00051826_51*, the marker associated with *Qph.ahau-1D.3* (Lv et al., 2024). *QPH.ynau-4D.2* is located approximately 22.66 Mb from *Xgwm192*, the nearest marker reported for *QPH.cgb-4D* (Wu et al., 2012). Among the other PH loci, *QPH.ynau-7B.1* lies within *QRC-7B-III*, whereas *QPH.ynau-6B.1* lies 2.447 Mb from *QRC-6B-I* (Xu et al., 2023). Although these QTLs did not overlap the detected selective sweep intervals, they provide additional targets for plant-height improvement alongside major dwarfing alleles. For *QPH.ynau-5B.1*, which overlapped selective sweep intervals, previous studies have confirmed that this chromosome harbors meta-QTL hotspots determining wheat plant architecture and yield components (Amalova et al., 2022; Kartseva et al., 2024; Lohani et al., 2026). The peak SNP of *QPH.ynau-5B.1* at 531.698 Mb lies within the 529.816–535.816 Mb PH interval reported previously and within regions associated with longest and shortest fertile-tiller length at 529.531–533.768 Mb and 531.656–533.749 Mb, respectively, in the worldwide panel (Li et al., 2022; Liu et al., 2023). *QPH.ynau-5B.1* localizes with major pleiotropic QTLs, which have also been reported (Amalova et al., 2022). The presence of an NAC domain-containing transcription factor within this interval provides a candidate for investigating the biological basis of the previously reported pleiotropic effects.

For SN, the two stable chromosome 1A loci showed positional relationships with previously reported regions associated with spike production. After matching coordinates between reference assemblies, the peak of *QSN.ynau-1A.2* was located at 36.777 Mb, within the 23.132–39.617 Mb effective-tiller-number interval (Zhang et al., 2024). *QSN.ynau-1A.4* mapped to 431.978 Mb, only 250.9 kb from *Tdurum_contig43203_318*, a marker associated with spike number measured in a 1-m row (Yang et al., 2020). These comparisons further support the relevance of these chromosome 1A regions to spike production. The peak of *QSN.ynau-7B.2* lies within the thousand-grain weight QTL interval (721.864–724.729 Mb). It is approximately 210.1 kb upstream of *TaSWEET6-7B* (*TraesCS7B03G1216700*), whose haplotypes were also associated with effective tiller number, supporting this gene as a candidate for *QSN.ynau-7B.2* (Jiao et al., 2023). No corresponding SN QTL was found for *QSN.ynau-2A.2* among the published loci examined. Its detection in the 2025–2026 trial and the BLUP analysis supports its potential use in the genetic improvement of SN. Additional comparisons showed that *QSN.ynau-1A.1* lies within the 28.6–30.7 Mb interval associated with thousand-kernel weight and spike length (Li et al., 2021). *QSN.ynau-2D.2* overlaps with the spikelet-density locus *QSD.sdau_2D.2*, with its peak only 0.282 Mb from the associated marker *Chr2D_577064215* (Niu et al., 2026). *QSN.ynau-5B.1* lies 0.507 Mb from the position of the spike-length locus *QSl.gaas-5B.1* (Guo et al., 2026). Previous meta-QTL analyses and GWAS targeting wheat yield components have highlighted the A sub-genome as a region determining tillering and effective SN (Li et al., 2019; Yang et al., 2020).

### 4.4 Selective sweep analyses in wheat improvement

Artificial selection during wheat improvement has reshaped genetic variation associated with agronomic performance and environmental adaptation (Cavanagh et al., 2013). In this study, selective sweep analyses using π ratio, *F_ST_*, and XP-CLR identified candidate genomic regions showing signatures consistent with breeding selection. Most genes within these regions were located in the A and B sub-genomes, whereas the D sub-genome contained fewer candidates. Previous resequencing of historical Chinese wheat cultivars revealed asymmetric selection among the three sub-genomes (Hao et al., 2020). Similarly, Li et al. (2022) reported an uneven distribution of selective sweeps, with regulatory genes on the A and B sub-genomes preferentially selected. These studies provide a broader context for the uneven distribution of candidate selected genes observed in our panel.

Integrating GWAS with selective sweep analysis identified overlapping regions at *QSN.ynau-1A.4*, *QPH.ynau-5B.1* and *QSN.ynau-6B.3*, providing candidates for investigating breeding-related changes in PH and SN. A previous study reported that more than half of 207 agronomic trait-associated loci overlapped genomic regions showing evidence of selection in Chinese and US wheat germplasm (Niu et al., 2023). The selected variants included both shared and region-specific components, indicating that wheat improvement has involved common breeding targets alongside locally differentiated genetic variation. The overlapping regions were further examined to prioritize positional candidate genes. *QPH.ynau-5B.1* contains a gene encoding an NAC domain-containing transcription factor, whereas *QSN.ynau-1A.4* encompasses genes encoding beta-glucanase and diacylglycerol kinase. These annotations suggest transcriptional regulation and metabolic processes as directions for investigating the biological basis of the overlapping signals. A combined analysis of GWAS and selective sweeps in Turkish winter wheat likewise identified genomic regions relevant to yield and adaptation (Sehgal et al., 2024). Cheng et al. (2024) demonstrated beneficial effects of selected landrace-derived alleles on agronomic traits after their introgression into a modern wheat genetic background. For the overlapping regions identified in this study, comparing haplotypes and their phenotypic effects could similarly help evaluate their potential for improving wheat architecture.

Although the major PH locus (*Rht-D1*) on chromosome 4D showed the strongest association with PH, it did not appear in the significantly selected intervals of the genome-wide selective sweep under the current detection threshold. The failure to detect a significant selection signature at this locus under these criteria may be attributed to several methodological and demographic factors. Selective sweep scans are inherently sensitive to marker coverage and density (especially on the D sub-genome), quality filtering criteria, and sliding-window specifications (Vitti et al., 2013). The frequency of the *AA* genotype at the representative SNP *4D_18894112* differed markedly between landraces and modern cultivars. This genotype, associated with reduced PH, occurred in 2 of 133 landraces (1.5%) and 37 of 147 modern cultivars (25.2%). Its enrichment in modern cultivars is consistent with breeding preferences for shorter plants, although population history and geographic composition may also contribute to this difference. Nevertheless, the *AA* genotype remained a minority in modern cultivars, indicating that the associated allele had not approached fixation in this panel. The coexistence of alternative genotypes in modern cultivars helps explain why allele-frequency differentiation was not accompanied by a pronounced local reduction in marker-based diversity.

## 5 Conclusions

By integrating high-density genome-wide variation maps with phenotypic data, this study characterized population structure and putative gene-flow patterns of the associated wheat panel and mapped 8 stable QTLs controlling PH or SN. Integrating GWAS with selective sweep analysis identified genomic regions in which reproducible trait associations overlapped signatures consistent with breeding selection. Functional annotation of genes within these regions provided positional candidates for investigating the genetic basis of PH and SN. These findings provide testable hypotheses and candidate targets for subsequent haplotype, expression, and functional analyses.

## Data Availability

The original contributions presented in the study are included in the article/**Supplementary Material**. Further inquiries can be directed to the corresponding author.
