# Supplementary material for: Genome-wide association study and selective sweep analyses identify loci associated with plant height and spike number in a Chinese micro-core collection of common wheat (Triticum aestivum L.)
Source: Front Plant Sci. 2026 Oct 30;17:2003113. doi: 10.3389/fpls.2026.2003113 (PMC13759778; doi:10.3389/fpls.2026.2003113)
Supplement: Supplementary file 1 [file DataSheet1.pdf]

# *Supplementary Material*

## 1 Supplementary Figures and Tables

### 1.1 Supplementary Figures

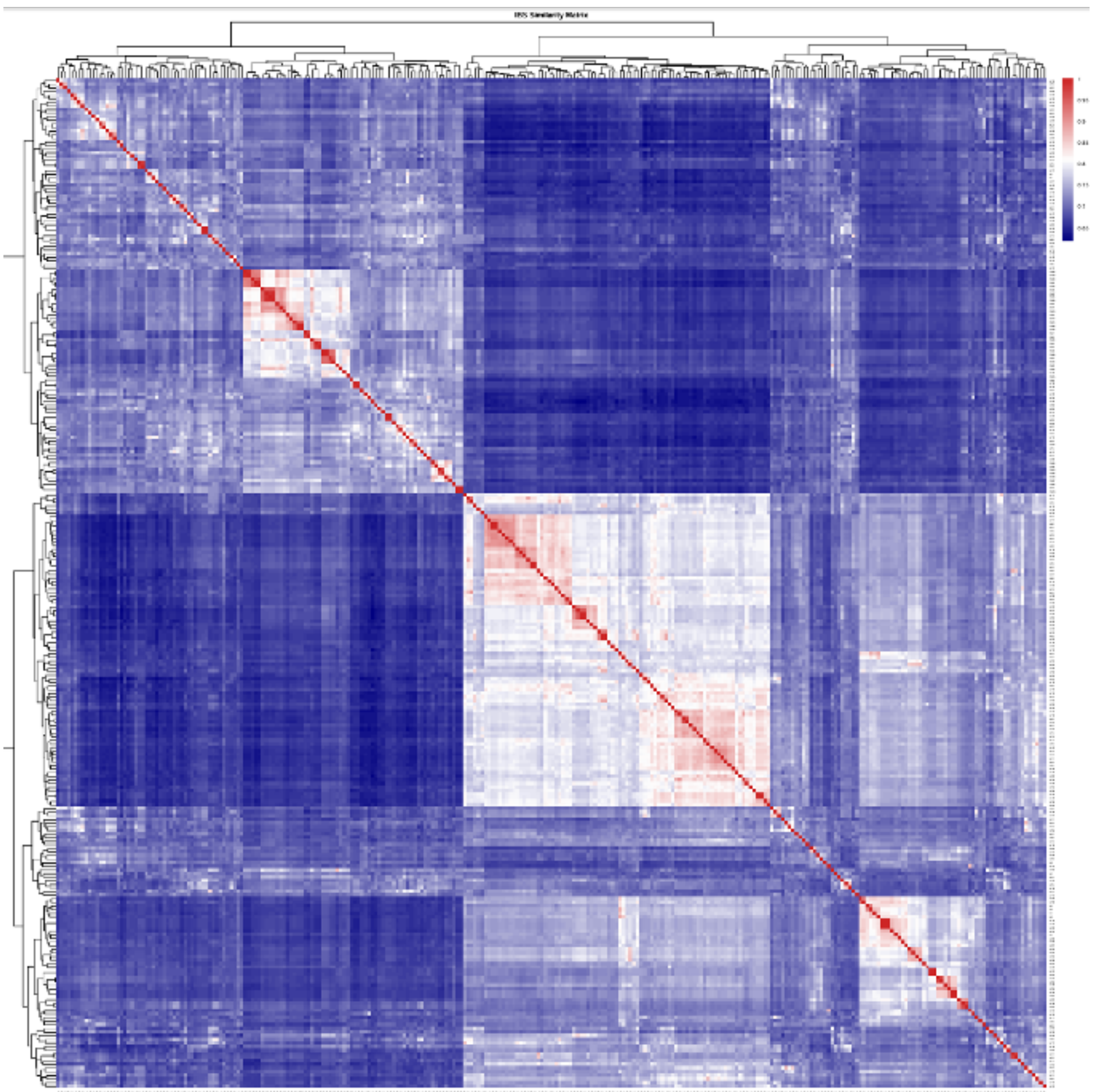

**Supplementary Figure S1** Genome-wide identity-by-state relationships among wheat accessions. Heatmap of pairwise identity-by-state genetic similarity among 280 wheat accessions based on 319,986 high-quality single-nucleotide polymorphism markers. Rows and columns represent accessions ordered by hierarchical clustering, as shown by the dendrograms; colors from blue to red indicate lower to higher genetic similarity.

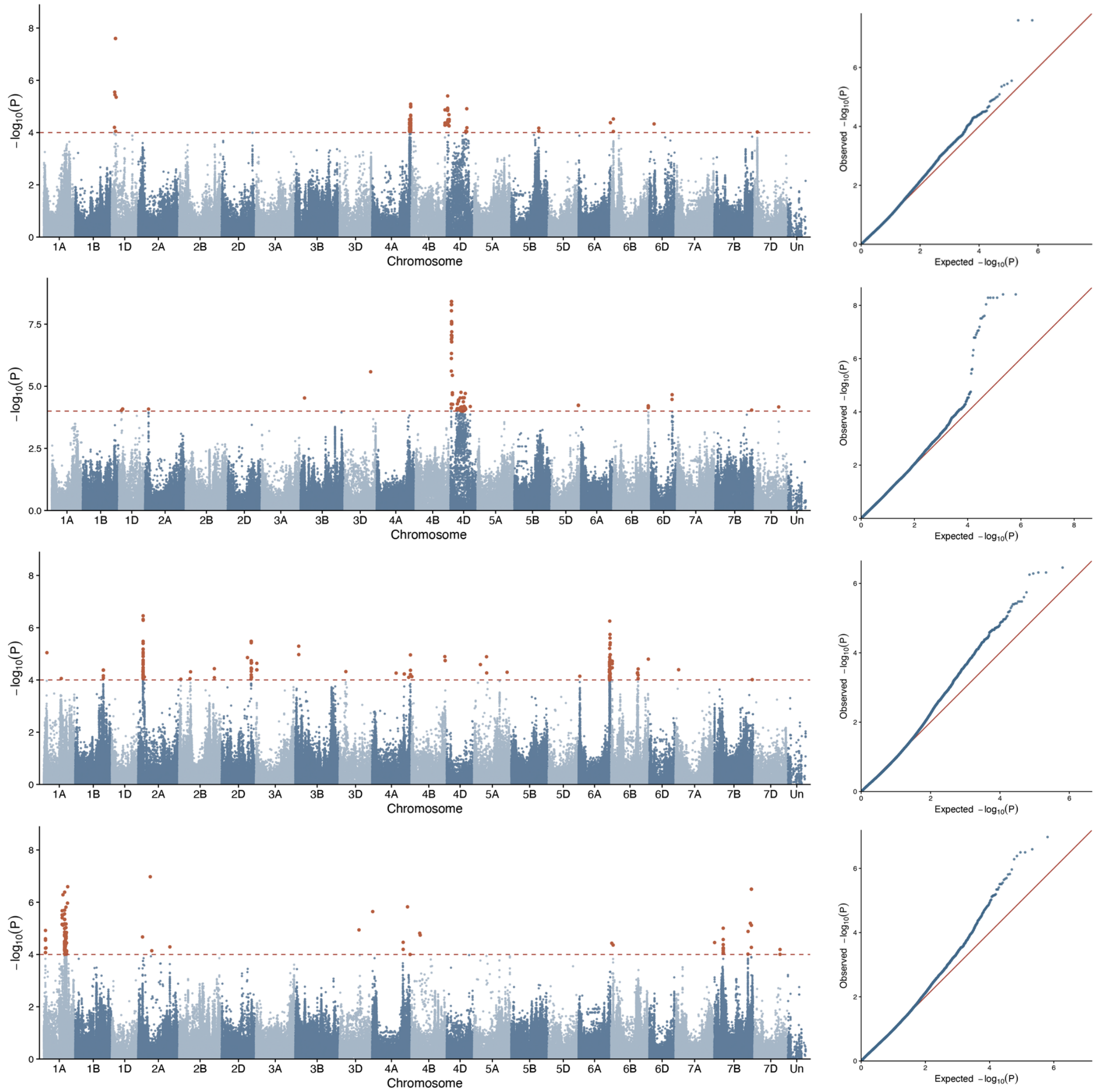


**Supplementary Figure S2** Association results for plant height and spike number in individual environments. Genome-wide association results for plant height (PH) and spike number per plant (SN). From top to bottom, the rows show SN2025, SN2026, PH2025, and PH2026; each row contains a Manhattan plot and a quantile-quantile (QQ) plot. In the Manhattan plots, chromosomes are shown in alternating colors, and the red line indicates $P = 10^{-4}$. In the QQ plots, blue points compare the observed and expected $-\log_{10}(P)$ values, and the red diagonal indicates the null expectation.

## 1.2 Supplementary Tables

**Supplementary Table S1.** QTLs for PH and SN detected across multiple datasets.

| Trait | Datasets | QTL | Peak SNP Position | *P*_Wald | MAF | BETA | PVE (%) |
|---|---|---|---|---|---|---|---|
| PH | 2025, 2026, BLUP | *QPH.ynau-1D.1* | *1D_67562322* | $2.51 \times 10^{-8}$ | 0.073 | -12.920 | 6.35 |
| PH | 2026 | *QPH.ynau-2A.1* | *2A_66538962* | $8.30 \times 10^{-5}$ | 0.297 | 5.665 | 3.47 |
| PH | 2026 | *QPH.ynau-3B.1* | *3B_76857171* | $2.96 \times 10^{-5}$ | 0.068 | 10.164 | 3.37 |
| PH | 2026 | *QPH.ynau-3D.1* | *3D_515096428* | $2.61 \times 10^{-6}$ | 0.061 | 10.992 | 3.76 |
| PH | 2025 | *QPH.ynau-4A.1* | *4A_751861561* | $8.16 \times 10^{-6}$ | 0.429 | -5.764 | 4.44 |
| PH | BLUP | *QPH.ynau-4B.1* | *4B_43533249* | $3.51 \times 10^{-5}$ | 0.080 | -7.807 | 3.36 |
| PH | 2025 | *QPH.ynau-4B.2* | *4B_653420946* | $1.35 \times 10^{-5}$ | 0.078 | -10.047 | 4.06 |
| PH | 2025, 2026, BLUP | *QPH.ynau-4D.1* | *4D_18894112* | $3.87 \times 10^{-9}$ | 0.143 | -10.986 | 8.21 |
| PH | 2025, 2026, BLUP | *QPH.ynau-4D.2* | *4D_389939803* | $2.10 \times 10^{-6}$ | 0.280 | 6.294 | 5.84 |
| PH | 2025 | *QPH.ynau-5B.1* | *5B_531698358* | $6.77 \times 10^{-5}$ | 0.282 | -8.898 | 9.12 |
| PH | 2026, BLUP | *QPH.ynau-5D.1* | *5D_532684498* | $2.55 \times 10^{-5}$ | 0.119 | -7.725 | 4.64 |
| PH | 2025 | *QPH.ynau-6A.1* | *6A_621277900* | $4.20 \times 10^{-5}$ | 0.357 | -5.337 | 3.54 |
| PH | 2025 | *QPH.ynau-6B.1* | *6B_40953486* | $3.02 \times 10^{-5}$ | 0.082 | 11.744 | 4.46 |
| PH | 2026 | *QPH.ynau-6B.2* | *6B_690097812* | $6.13 \times 10^{-5}$ | 0.160 | -6.325 | 2.75 |
| PH | 2025 | *QPH.ynau-6D.1* | *6D_91076733* | $4.68 \times 10^{-5}$ | 0.164 | -6.386 | 2.96 |
| PH | 2026 | *QPH.ynau-6D.2* | *6D_415448413* | $2.19 \times 10^{-5}$ | 0.314 | -6.005 | 4.09 |
| PH | 2026 | *QPH.ynau-7B.1* | *7B_715673809* | $9.09 \times 10^{-5}$ | 0.444 | 4.560 | 2.68 |
| PH | 2026 | *QPH.ynau-7D.1* | *7D_470827335* | $6.79 \times 10^{-5}$ | 0.357 | 5.040 | 2.98 |
| SN | 2026 | *QSN.ynau-1A.1* | *1A_29402108* | $1.20 \times 10^{-5}$ | 0.480 | 0.514 | 8.25 |
| SN | 2026, BLUP | *QSN.ynau-1A.2* | *1A_39056365* | $1.50 \times 10^{-6}$ | 0.080 | 0.107 | 7.68 |
| SN | 2025 | *QSN.ynau-1A.3* | *1A_57720702* | $9.05 \times 10^{-6}$ | 0.292 | 0.619 | 8.40 |
| SN | 2026, BLUP | *QSN.ynau-1A.4* | *1A_433811958* | $7.75 \times 10^{-8}$ | 0.330 | -0.096 | 18.07 |
| SN | BLUP | *QSN.ynau-1B.1* | *1B_554536510* | $7.06 \times 10^{-6}$ | 0.155 | -0.089 | 9.75 |
| SN | 2025 | *QSN.ynau-2A.1* | *2A_95311924* | $3.50 \times 10^{-7}$ | 0.167 | -0.764 | 8.57 |
| SN | 2026, BLUP | *QSN.ynau-2A.2* | *2A_234772036* | $1.06 \times 10^{-7}$ | 0.052 | 1.295 | 11.30 |
| SN | 2025 | *QSN.ynau-2B.1* | *2B_684542449* | $3.70 \times 10^{-5}$ | 0.075 | 0.866 | 5.21 |
| SN | 2025 | *QSN.ynau-2D.1* | *2D_503856699* | $1.39 \times 10^{-5}$ | 0.123 | -0.717 | 5.81 |
| SN | 2025 | *QSN.ynau-2D.2* | *2D_577346281* | $3.32 \times 10^{-6}$ | 0.053 | 1.189 | 6.76 |
| SN | 2025 | *QSN.ynau-3A.1* | *3A_15986085* | $2.31 \times 10^{-5}$ | 0.220 | -0.595 | 6.36 |
| SN | 2025 | *QSN.ynau-3B.1* | *3B_66033710* | $5.11 \times 10^{-6}$ | 0.060 | -0.991 | 6.03 |
| SN | 2026 | *QSN.ynau-3D.1* | *3D_376917629* | $1.15 \times 10^{-5}$ | 0.073 | 0.917 | 7.45 |
| SN | BLUP | *QSN.ynau-3D.2* | *3D_441460901* | $3.71 \times 10^{-5}$ | 0.209 | 0.074 | 8.30 |
| SN | BLUP | *QSN.ynau-4A.1* | *4A_49909233* | $5.32 \times 10^{-5}$ | 0.295 | 0.067 | 8.13 |
| SN | 2025 | *QSN.ynau-4A.2* | *4A_627448016* | $5.92 \times 10^{-5}$ | 0.093 | 0.775 | 5.21 |

| Trait | Datasets | QTL | Peak SNP Position | *P*_Wald | MAF | BETA | PVE (%) |
|---|---|---|---|---|---|---|---|
| SN | 2026 | *QSN.ynau-4A.3* | *4A_690648558* | $1.50 \times 10^{-6}$ | 0.065 | 1.087 | 8.63 |
| SN | 2025 | *QSN.ynau-4A.4* | *4A_713366130* | $7.97 \times 10^{-5}$ | 0.301 | -0.525 | 6.09 |
| SN | 2026 | *QSN.ynau-4B.1* | *4B_159413257* | $1.53 \times 10^{-5}$ | 0.054 | -0.977 | 6.29 |
| SN | 2025 | *QSN.ynau-4B.2* | *4B_649990735* | $1.28 \times 10^{-5}$ | 0.056 | 1.025 | 5.41 |
| SN | 2025 | *QSN.ynau-4B.3* | *4B_656694968* | $1.81 \times 10^{-5}$ | 0.070 | -0.955 | 5.76 |
| SN | BLUP | *QSN.ynau-4D.1* | *4D_82422866* | $5.95 \times 10^{-5}$ | 0.064 | 0.128 | 8.95 |
| SN | 2025 | *QSN.ynau-5A.1* | *5A_242858034* | $1.29 \times 10^{-5}$ | 0.275 | -0.861 | 15.73 |
| SN | BLUP | *QSN.ynau-5A.2* | *5A_700057864* | $2.92 \times 10^{-6}$ | 0.113 | 0.085 | 6.35 |
| SN | BLUP | *QSN.ynau-5B.1* | *5B_400839024* | $1.87 \times 10^{-5}$ | 0.071 | -0.092 | 4.96 |
| SN | 2025 | *QSN.ynau-6A.1* | *6A_607832023* | $5.60 \times 10^{-7}$ | 0.088 | 0.971 | 8.00 |
| SN | 2025 | *QSN.ynau-6A.2* | *6A_613147597* | $1.81 \times 10^{-6}$ | 0.155 | -0.795 | 7.21 |
| SN | 2025 | *QSN.ynau-6B.1* | *6B_18952245* | $1.95 \times 10^{-5}$ | 0.094 | -0.849 | 6.55 |
| SN | 2026 | *QSN.ynau-6B.2* | *6B_36557620* | $4.36 \times 10^{-5}$ | 0.217 | 0.558 | 6.60 |
| SN | 2025 | *QSN.ynau-6B.3* | *6B_525579060* | $3.80 \times 10^{-5}$ | 0.076 | 0.857 | 5.71 |
| SN | 2025 | *QSN.ynau-6B.4* | *6B_724444010* | $1.60 \times 10^{-5}$ | 0.054 | -1.462 | 5.79 |
| SN | 2025 | *QSN.ynau-7A.1* | *7A_59731958* | $4.07 \times 10^{-5}$ | 0.164 | -0.734 | 8.08 |
| SN | 2026 | *QSN.ynau-7B.1* | *7B_171724668* | $9.88 \times 10^{-6}$ | 0.247 | 0.648 | 8.98 |
| SN | 2026, BLUP | *QSN.ynau-7B.2* | *7B_722662852* | $3.17 \times 10^{-7}$ | 0.085 | 0.990 | 9.65 |
| SN | 2026 | *QSN.ynau-7D.1* | *7D_503233497* | $6.45 \times 10^{-5}$ | 0.147 | -0.591 | 5.57 |

**Supplementary Table S2.** Functional annotation of genes within candidate selective sweep regions in common wheat.

| Gene ID | Chr. | Gene start (bp) | Gene end (bp) | Strand | Description |
|---|---|---|---|---|---|
| *TraesCS1A03G0241600* | chr1A | 98053366 | 98058818 | + | DnaJ protein ERDJ3B |
| *TraesCS1A03G0242100* | chr1A | 98087498 | 98093734 | - | Dihydrolipoyl dehydrogenase |
| *TraesCS1A03G0244300* | chr1A | 98713474 | 98715330 | - | Pentatricopeptide repeat-containing protein At3g05340 |
| *TraesCS1A03G0244400* | chr1A | 98718145 | 98727014 | - | EMB3117 |
| *TraesCS1A03G0602600* | chr1A | 407160135 | 407165504 | - | Thiamine pyrophosphokinase |
| *TraesCS1A03G0607900* | chr1A | 411458989 | 411459864 | + | rRNA N-glycosidase |
| *TraesCS1A03G0608100* | chr1A | 411460021 | 411462857 | - | PrfB3 |
| *TraesCS1A03G0608300* | chr1A | 411775141 | 411779297 | + | beta glucanase |
| *TraesCS1A03G0610900* | chr1A | 417178525 | 417183175 | + | Diacylglycerol kinase |
| *TraesCS1A03G0622600* | chr1A | 428379187 | 428382400 | + | Probable ADP-ribosylation factor GTPase-activating protein AGD11 |
| *TraesCS1A03G0623300* | chr1A | 428735753 | 428737777 | + | Annexin |
| *TraesCS1A03G0623400* | chr1A | 428737666 | 428740933 | - | Mitochondrial fission 1 protein |
| *TraesCS1A03G0623600* | chr1A | 428778226 | 428782416 | - | AP-3 complex subunit mu |
| *TraesCS1A03G0757000* | chr1A | 499087711 | 499088987 | - | Peroxidase |
| *TraesCS1A03G0757100* | chr1A | 499092963 | 499093940 | - | Peroxidase |
| *TraesCS1A03G0758200* | chr1A | 499369457 | 499375225 | - | Protein DETOXIFICATION |
| *TraesCS1A03G0760300* | chr1A | 499644013 | 499647429 | + | NADH-cytochrome b5 reductase |
| *TraesCS1A03G0760700* | chr1A | 499682261 | 499685682 | - | GPN-loop GTPase |
| *TraesCS1A03G0761400* | chr1A | 499954374 | 499957004 | - | Similar to ADP-ribosylation factor 1 |
| *TraesCS1A03G0902000* | chr1A | 550220815 | 550222665 | - | E3 ubiquitin-protein ligase |
| *TraesCS2A03G0253500* | chr2A | 77428562 | 77432705 | + | Cellulose synthase-like protein D4 |
| *TraesCS2A03G0348000* | chr2A | 125295549 | 125296459 | - | Dirigent protein |
| *TraesCS2A03G0348600* | chr2A | 125400236 | 125401004 | - | Dirigent protein |
| *TraesCS2A03G0348800* | chr2A | 125703870 | 125704388 | - | Dirigent protein |
| *TraesCS2A03G0349200* | chr2A | 125763648 | 125768167 | - | Sucrose synthase |
| *TraesCS2A03G0349300* | chr2A | 125833844 | 125836307 | + | 40S ribosomal protein SA |
| *TraesCS2A03G0354400* | chr2A | 129841173 | 129844358 | + | Proteasome subunit alpha type |
| *TraesCS2A03G0355500* | chr2A | 130539398 | 130548239 | - | Exportin-T |
| *TraesCS2A03G0360700* | chr2A | 135294426 | 135297486 | + | Pectinesterase |

| Gene ID | Chr. | Gene start (bp) | Gene end (bp) | Strand | Description |
|---|---|---|---|---|---|
| *TraesCS2A03G0362900* | chr2A | 137422986 | 137440104 | - | APETALA1 (AP1)/ FRUITFULL (FUL)-like MADS box transcription factor |
| *TraesCS2A03G0363200* | chr2A | 137903996 | 137907304 | - | UDP-arabinopyranose mutase |
| *TraesCS2A03G0364600* | chr2A | 138223128 | 138225267 | + | COBRA-like protein |
| *TraesCS2A03G0364700* | chr2A | 138227090 | 138230888 | - | COBRA-like protein |
| *TraesCS2A03G0365400* | chr2A | 138257200 | 138261152 | - | Probable 6-phosphogluconolactonase 1 |
| *TraesCS2A03G0368800* | chr2A | 140878115 | 140887128 | - | Histone deacetylase |
| *TraesCS2A03G0382700* | chr2A | 149303328 | 149308126 | + | Adenylyl-sulfate kinase |
| *TraesCS2A03G0382900* | chr2A | 149491323 | 149499388 | + | Protein FREE1 |
| *TraesCS2A03G0388100* | chr2A | 153483263 | 153486748 | + | Ubiquitin-conjugating enzyme COP10 |
| *TraesCS2A03G0388400* | chr2A | 153498658 | 153500214 | + | Chlorophyll a-b binding protein, chloroplastic |
| *TraesCS2A03G0457400* | chr2A | 203645210 | 203650363 | + | Peptide chain release factor PrfB1, chloroplastic |
| *TraesCS2A03G0458700* | chr2A | 204718211 | 204722710 | - | Similar to Chitin-inducible gibberellin-responsive protein |
| *TraesCS2A03G0458900* | chr2A | 205157049 | 205165070 | + | Formin-like protein |
| *TraesCS2A03G0459100* | chr2A | 205163766 | 205165067 | - | Protein H2A.7 |
| *TraesCS2A03G0459500* | chr2A | 205456594 | 205457659 | - | Histone H2A |
| *TraesCS2A03G0460500* | chr2A | 206580844 | 206584312 | - | Beta-amylase |
| *TraesCS2A03G0461100* | chr2A | 206599879 | 206602993 | - | Beta-amylase |
| *TraesCS2A03G0760900* | chr2A | 529939639 | 529941012 | + | PsbP domain-containing protein 2, chloroplastic |
| *TraesCS2A03G0762300* | chr2A | 530219848 | 530221638 | - | Glutathione S-transferase TCHQD |
| *TraesCS2A03G0763400* | chr2A | 531330887 | 531334220 | - | Protein disulfide-isomerase |
| *TraesCS2A03G0763600* | chr2A | 531339529 | 531342209 | - | Pentatricopeptide repeat-containing protein At3g26540 |
| *TraesCS2B03G0815100* | chr2B | 456564790 | 456565511 | + | Putative membrane protein |
| *TraesCS2B03G0815300* | chr2B | 456597548 | 456602058 | + | Putative pentatricopeptide repeat-containing protein At5g37570 |
| *TraesCS2B03G0816500* | chr2B | 456956453 | 456958080 | + | HVA22-like protein |
| *TraesCS2B03G0816700* | chr2B | 457172699 | 457173568 | + | Similar to H0622F05.9 protein |

| Gene ID | Chr. | Gene start (bp) | Gene end (bp) | Strand | Description |
|---|---|---|---|---|---|
| *TraesCS2B03G0904100* | chr2B | 504755525 | 504758779 | - | Pentatricopeptide repeat-containing protein At3g63370, chloroplastic |
| *TraesCS3A03G0522000* | chr3A | 358222427 | 358227273 | - | Vacuolar cation/proton exchanger |
| *TraesCS3B03G1131800* | chr3B | 716328162 | 716331394 | + | Expressed protein 1 |
| *TraesCS3B03G1131900* | chr3B | 716334893 | 716336066 | - | Cysteine-rich peptide |
| *TraesCS3B03G1132000* | chr3B | 716336746 | 716342131 | - | Zinc finger protein |
| *TraesCS5A03G1291000* | chr5A | 708554813 | 708565975 | + | Protein DETOXIFICATION |
| *TraesCS5A03G1295800* | chr5A | 710066497 | 710070348 | - | Beta-amylase |
| *TraesCS5B03G0892000* | chr5B | 538298830 | 538301027 | + | Protein DETOXIFICATION |
| *TraesCS5B03G0893200* | chr5B | 538871547 | 538873699 | + | NAC domain containing protein 57 |
| *TraesCS5B03G0893500* | chr5B | 538886711 | 538887286 | - | Histone H4 variant TH011 |
| *TraesCS5D03G1150800* | chr5D | 548226694 | 548227914 | + | Expansin |
| *TraesCS5D03G1150900* | chr5D | 548244492 | 548245794 | + | Expansin |
| *TraesCS5D03G1151900* | chr5D | 548698627 | 548700240 | - | Glycosyltransferase |
| *TraesCS5D03G1152000* | chr5D | 548709991 | 548712164 | - | Patatin |
| *TraesCS5D03G1152200* | chr5D | 548732230 | 548734341 | + | Patatin |
| *TraesCS5D03G1152500* | chr5D | 548793862 | 548795550 | + | 3-ketoacyl-CoA synthase |
| *TraesCS5D03G1158400* | chr5D | 550231324 | 550236910 | - | Pre-mRNA processing factor |
| *TraesCS5D03G1158700* | chr5D | 550267298 | 550269718 | + | Serine/threonine-protein kinase |
| *TraesCS5D03G1160000* | chr5D | 550496913 | 550501544 | + | Serine/threonine protein phosphatase 2A regulatory subunit |
| *TraesCS7A03G0299600* | chr7A | 85136145 | 85139165 | - | Serine/threonine-protein phosphatase |
| *TraesCS7A03G0300600* | chr7A | 85403320 | 85405698 | - | Serine/threonine-protein kinase |
| *TraesCS7A03G0300700* | chr7A | 85411337 | 85413778 | - | Serine/threonine-protein kinase |
| *TraesCS7A03G0303400* | chr7A | 85826749 | 85840265 | - | Histone-lysine N-methyltransferase |
| *TraesCS7A03G0303800* | chr7A | 86135954 | 86146286 | - | Histone-lysine N-methyltransferase |
| *TraesCS7D03G0959100* | chr7D | 527225655 | 527229720 | + | high chlorophyll fluorescence phenotype 173 |
| *TraesCS7D03G0959200* | chr7D | 527556462 | 527563650 | + | Poly [ADP-ribose] polymerase |
| *TraesCS7D03G0959500* | chr7D | 527611329 | 527616286 | + | Delta-aminolevulinic acid dehydratase |

| Gene ID | Chr. | Gene start (bp) | Gene end (bp) | Strand | Description |
|---|---|---|---|---|---|
| *TraesCS7D03G0974200* | chr7D | 532902444 | 532908934 | - | Similar to P1B-type heavy metal transporting ATPase |
